# Case Report: Papillophlebitis associated with MOG-IgG-associated optic neuritis

**DOI:** 10.3389/fmed.2025.1664151

**Published:** 2025-09-29

**Authors:** Yuanmin Dai, Siran Zhang, Yue Lou

**Affiliations:** ^1^Department of Ophthalmology, Zhejiang Hospital, Hangzhou, Zhejiang, China; ^2^Department of Neurology, Zhejiang Hospital, Hangzhou, Zhejiang, China

**Keywords:** encephalitis, optic neuritis, central retinal vein occlusion, papillophlebitis, ranibizumab

## Abstract

Optic neuritis is a common manifestation in patients with myelin oligodendrocyte glycoprotein antibody-associated disease (MOGAD). However, its association with papillophlebitis is rare and has only been sporadically reported in the literature. Papillophlebitis is primarily characterized by central retinal vein occlusion (CRVO) and typically affects young to middle-aged adults. We present the case of a 27-year-old woman who initially presented to the emergency department with headache, fever, and seizures. During her hospitalization, MOGAD was diagnosed based on brain MRI, cerebrospinal fluid analysis, and serological testing. Following the initiation of systemic corticosteroid therapy, bilateral optic neuritis was observed. One month later, the patient reported decreased vision in her left eye. Multimodal retinal imaging revealed tortuous retinal veins, flame-shaped hemorrhages, macular edema, and mild leakage from the optic disk and retinal vessels on fluorescein angiography (FA). Genetic testing identified homozygous MTHFR (C665T) and heterozygous PAI-1 (4G/5G) mutations, which supported the diagnosis of papillophlebitis. The patient’s vision improved following intravitreal anti-VEGF therapy. To the best of our knowledge, this is the first reported case of papillophlebitis occurring as a secondary complication of MOG-associated optic neuritis. This finding provides new insight into the spectrum of MOGAD and underscores the importance of regular fundus examinations during treatment to facilitate the timely management of ocular complications.

## Introduction

Myelin oligodendrocyte glycoprotein antibody-associated encephalitis (MOGAD) is an inflammatory demyelinating disease of the central nervous system (CNS), characterized by immune-mediated inflammation affecting the optic nerves, brain, and spinal cord. The clinical manifestations of MOGAD are diverse, primarily including optic neuritis (ON), optic perineuritis, acute disseminated encephalomyelitis (ADEM), transverse myelitis (TM), cortical encephalitis, and the involvement of the brainstem or cerebellum (1, 2). Glucocorticoid therapy is the primary treatment approach for this condition, demonstrating efficacy in improving clinical outcomes. In patients with MOGAD, optic neuritis is a prevalent manifestation, observed in over 50% of cases (3).

To date, several uncommon ophthalmic complications associated with MOG-associated optic neuritis have been documented, including uveitis, retinal vasculitis, retinal vein occlusion, preretinal hemorrhage, optic disk ischemia, and others (4–10). These complications, although rare, have predominantly been reported in case studies, providing valuable insights into the clinical spectrum and diagnostic considerations of this disease. In this report, we present a rare case of a patient initially diagnosed with MOGAD who developed bilateral optic neuritis and unilateral papillophlebitis during the course of treatment.

## Case presentation

A 27-year-old woman presented with a 2-week history of paroxysmal, needle-prick-like headaches accompanied by low-grade fever, with her body temperature consistently measuring approximately 37.5 °C. The initial evaluation included a plain head computed tomography (CT) scan, which yielded normal findings. The patient was prescribed indomethacin and cefdinir for symptomatic management. However, after more than 10 days of medication adherence, her headache and fever showed only minimal improvement. Three hours before being hospitalized, the patient experienced a seizure while on a bus, characterized by loss of consciousness, frothing at the mouth, and leftward eye deviation. The episode lasted approximately 20 s before resolving spontaneously. Following the event, the patient remained in a state of confusion. She had no family history of neurological or ophthalmological disorders.

**Figure 1:**
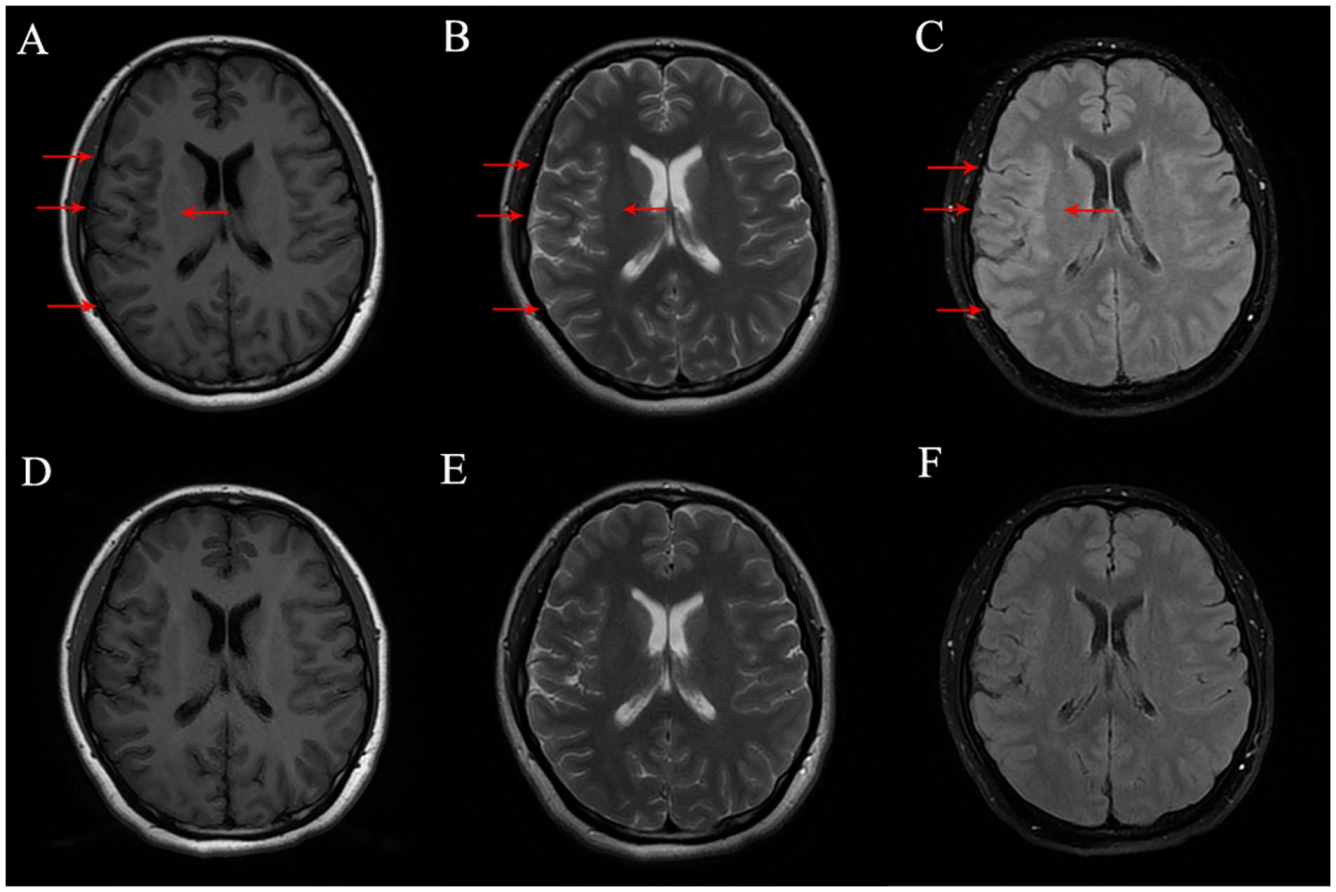
**(A–C)** Brain MR on Tl FLAIR image, T2-weighted image and T2 FLAIR image before treatment, respectively, and the lesion was marked with red arrow. **(D–F)** Brain MR on Tl FLAIR image, T2-weighted image and T2 FLAIR Image six-month after treatment, respectively.

Physical examination revealed non-fluent speech, and muscle strength in the left upper limb was graded at 1. Both the Babinski sign and the Chaddock sign were negative. Brain magnetic resonance imaging (MRI) revealed hypointensity on T1-weighted images and hyperintensity on T2-weighted and fluid-attenuated inversion recovery (FLAIR) sequences in the right frontal and parietal sulci. There was slight thickening of the cortex in the right frontal, parietal, and temporal lobes. Enhanced brain MRI showed no marked enhancement (Figure 1). To assess for spinal cord involvement, a thoracic spine MRI was performed, which returned normal results. Due to the patient’s financial constraints and the absence of symptoms indicating cervical and lumbar spinal cord involvement, MRI of the cervical and lumbar spine was not conducted. Blood test results showed a leukocyte count of 14.6 × 10^9^/L, with neutrophils accounting for 79.7%. The erythrocyte sedimentation rate (ESR) was 27 mm/h, the folic acid level was 6.07 mmol/L, the fibrinogen level was 5.65 g/L, the prothrombin time (PT) was 15 s, the triglyceride levels were 2.57 mmol/L, and the total cholesterol levels were 7.08 mmol/L. Cerebrospinal fluid (CSF) analysis revealed a slightly increased white cell count of 15 × 10^6^/L and glucose levels of 4.9 mmol/L, with normal protein and chloride levels. MOG-IgG was detected in both CSF (titer: 1:10) and serum (titer: 1:32). Aquaporin-4 antibodies, autoimmune encephalitis antibodies, and paraneoplastic antibodies were negative in both serum and CSF. Vitamin B12, homocysteine, thyroid hormones, related antibodies, and tumor markers were within normal limits. Additional autoimmune, infectious, and metabolic evaluations yielded negative results.

**Figure 2:**
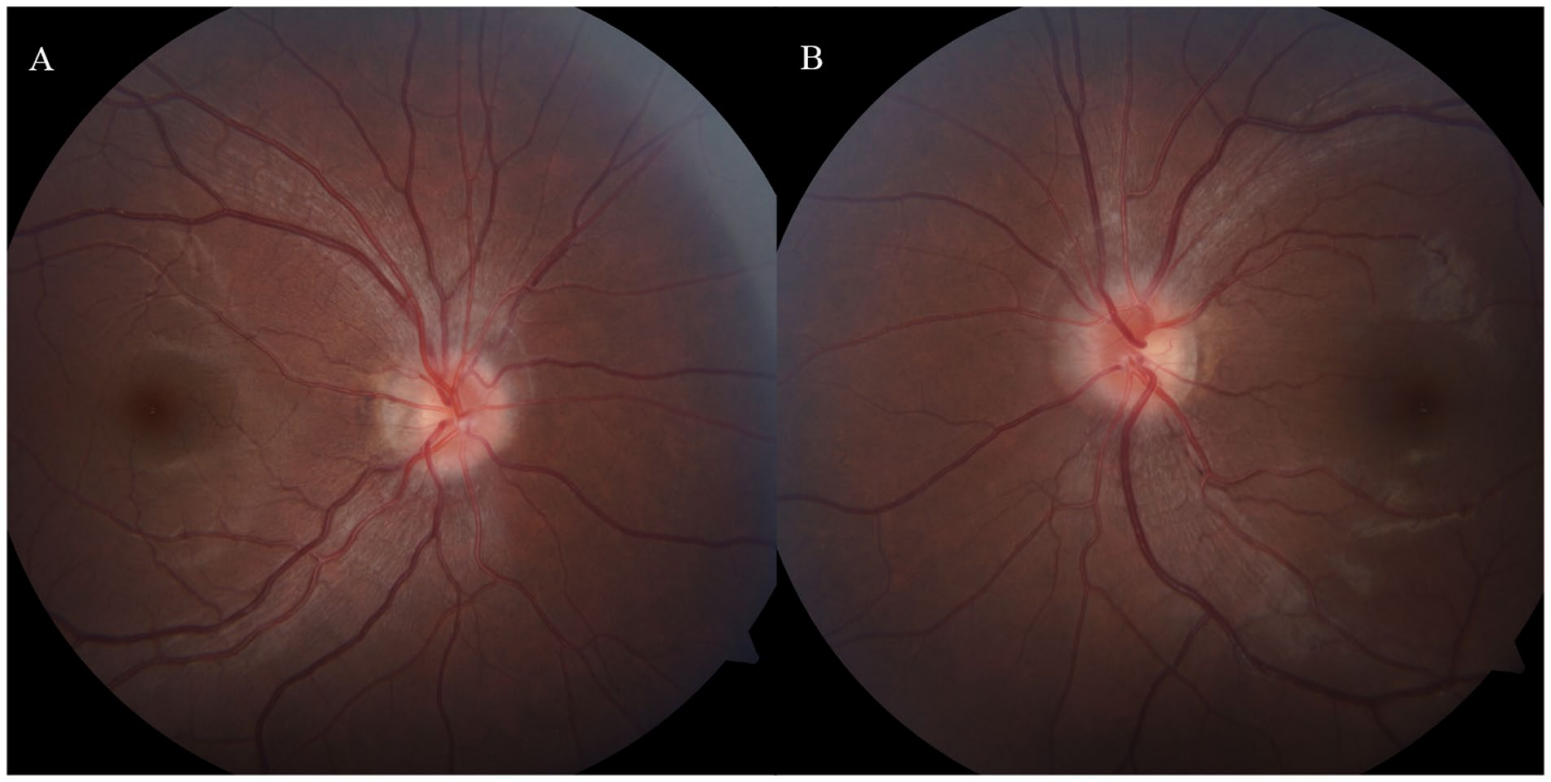
**(A,B)** Bilateral optic disk edema with indistinct margins and marked congestion of the left optic disk, MOG-IgG-associate ON was diagnosed during the hospitalization.

**Figure 3:**
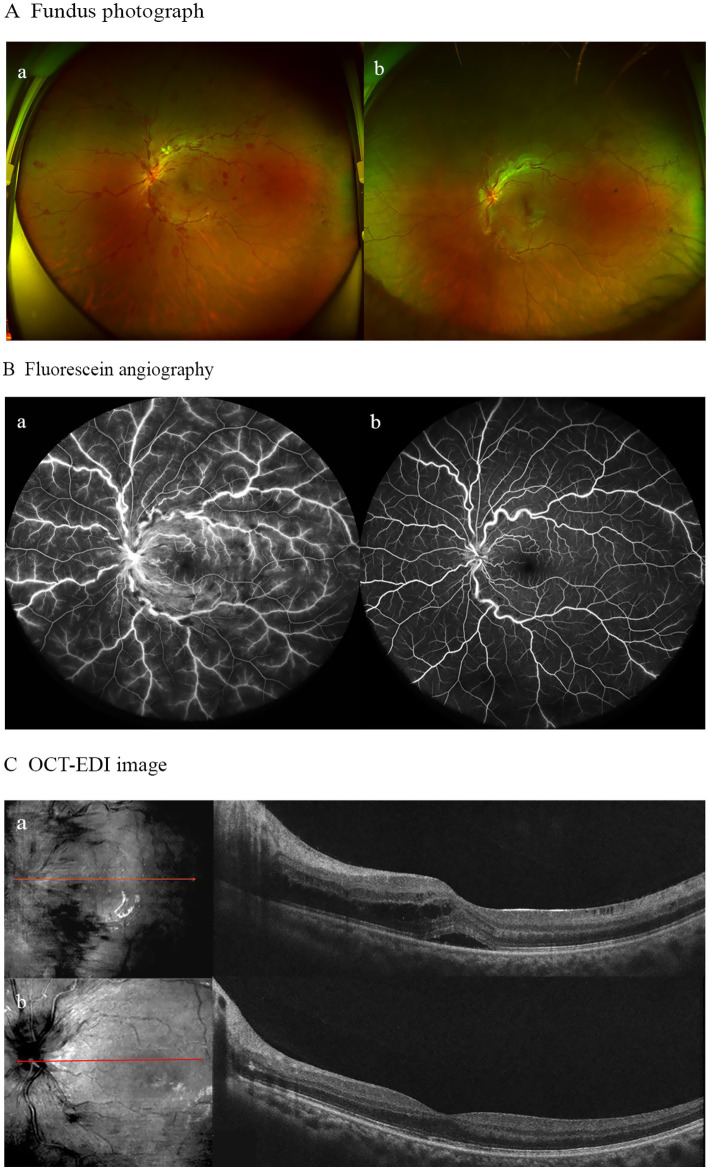
**(A)** Fundus photograph ofthe left eye demonstrating optic disk edema, tortuous veins, retinal hemorrhage, and cotton wool spots in the posterior pole before treatment (a); with residual slight optic disk edema, tortuous veins, and retinal hemorrhage after treatment (b). **(B)** Fluorescein angiography revealed vascular/optic nerve staining and mild macular leakage prior to treatment (a), showing no significant vascular leakage post-treatment (b). **(C)** OCT-EDI imagmg demonstrated subfoveal fluid with retinal thickening at baseline (a), followed by resolution of macular edema and complete absorption of subretinal fluid after 6 months of follow-up (b).

Due to the initial suspicion of viral encephalitis, the neurologist administered intravenous ganciclovir antiviral therapy at a dosage of 0.25 g twice daily for 3 days. Once MOG-IgG-associated encephalitis was diagnosed, the treatment plan was adjusted to include intravenous immunoglobulin at a dose of 0.4 g/kg per day for 5 days, along with intravenous methylprednisolone at 80 mg per day for 5 days. Following the completion of the intravenous methylprednisolone course, the patient was transitioned to oral prednisone at a dosage of 32 mg per day, with a tapering schedule of 4 mg per week. During hospitalization, the patient denied any ocular symptoms; however, a comprehensive ophthalmological consultation revealed a positive relative afferent pupillary defect (RAPD), bilateral optic disk edema, and marked congestion of the left optic disk. The patient’s best-corrected visual acuity (BCVA) was measured at 20/20 in both eyes (Figure 2). One month later, the patient presented to the ophthalmology department with a decline in vision in her left eye. At that time, BCVA was 15/20 in the left eye and 20/20 in the right eye, with an intraocular pressure of 15 mmHg. Fundoscopic examination revealed optic disk edema, tortuous veins, retinal hemorrhage, and cotton wool spots in the posterior pole of the left eye, while no pathological changes were noted in the right eye. Optical coherence tomography of the macula showed subfoveal fluid accumulation and retinal thickening (350 μm) in the macular region of the left eye. Fluorescein angiography indicated normal retinal circulation time, dilated retinal vessels, and vascular and optic nerve staining, as well as mild macular leakage in the left eye (Figure 3). Visual field testing and RAPD examination were within normal limits. Based on the clinical symptoms and multimodal imaging findings, a diagnosis of papillophlebitis was made. Further genetic testing revealed homozygous MTHFR (C665T) and heterozygous PAI-1 (4G/5G) mutations.

During the 6-month follow-up, the patient achieved complete recovery. A brain MRI scan showed resolution of the encephalitis. In terms of ophthalmic treatment outcomes, improvements in macular edema and visual acuity were observed following three injections of ranibizumab (Figure 3).

## Discussion

In this case report, we present a young female patient who developed left-eye papillophlebitis after being diagnosed with myelin oligodendrocyte glycoprotein antibody disease (MOGAD). At the time of MOGAD confirmation, the patient already exhibited clinical signs of optic neuritis. One month after starting corticosteroid treatment, she experienced a moderate decline in visual acuity in her left eye. Fundus angiography (FA) examinations revealed tortuous retinal veins and mild vascular leakage. Notably, the patient had no pre-existing systemic risk factors for retinal vascular disease. Serological tests indicated an abnormal prothrombin time (PT) and folic acid levels, while genetic testing revealed homozygous mutations in the methylenetetrahydrofolate reductase (MTHFR) gene (C665T) and heterozygous mutations in the plasminogen activator inhibitor-1 (PAI-1) gene (4G/5G). Additional serological and cerebrospinal fluid (CSF) tests ruled out vasculitis-related disorders and other autoimmune diseases.

Papillophlebitis commonly manifests with central retinal vein occlusion (CRVO), the second most prevalent retinal vascular disease. It typically occurs in middle-aged and older patients and is often associated with systemic conditions, including hypertension, hyperlipidemia, and hyperglycemia. Arteriosclerosis is the most common underlying cause of CRVO, while other contributing factors include venous wall pathologies (e.g., vasculitis), vascular compression, and hemodynamic alterations (11–13). Papillophlebitis, however, is considered a form of CRVO that occurs in younger individuals and is frequently linked to inflammatory or autoimmune diseases. In this case, the clinical presentation, imaging findings, and genetic test results were more consistent with papillophlebitis.

Although optic neuritis is a common manifestation of MOGAD, the development of secondary ophthalmic complications is relatively infrequent but clinically significant. While previous studies have reported various ophthalmic complications associated with MOG-associated optic neuritis, papillophlebitis has not been documented in this context prior to the present case. Papillophlebitis has been observed in conjunction with systemic and ocular inflammatory conditions, including polyarteritis nodosa, Guillain–Barré syndrome, Buerger’s disease, ulcerative colitis, and Eales’ disease (14–17). The precise pathogenesis of papillophlebitis remains elusive, but it is believed to involve a multitude of factors, including immune reactions, infections, and hyperlipidemia (18, 19).

Thrombophilia has been identified as a potential underlying cause of papillophlebitis in several studies, encompassing mutations such as Factor V Leiden (FVL) and methylenetetrahydrofolate reductase (MTHFR-C677T), as well as conditions such as hyperhomocysteinemia and deficiencies in vitamin B6, folic acid, or proteins C and S (20–22). In the present case, the patient exhibited typical clinical manifestations of papillophlebitis along with characteristic findings on multimodal retinal imaging, collectively supporting the diagnosis of papillophlebitis associated with MOG-associated optic neuritis. The homozygous MTHFR (C665T) mutation disrupts folic acid metabolism, leading to elevated homocysteine levels, impaired vascular wall integrity, and an increased risk of thrombosis. Similarly, the heterozygous PAI-1 (4G/5G) mutation reduces fibrinolytic capacity, further enhancing the risk of thrombotic events. The combination of these two mutations significantly increases the likelihood of thrombotic complications, providing critical diagnostic evidence for this case. Additionally, the patient’s retinal findings were strikingly similar to those of venous stasis retinopathy (VSR), a condition that has been previously reported as an ophthalmic complication of MOG-associated optic neuritis (9, 10).

Venous stasis retinopathy (VSR), characterized by incomplete occlusion of the central retinal vein, represents an early form of ocular ischemia, as initially described by Hayreh (19). It is marked by central retinal vein occlusion with clinical features such as severe visual acuity decline and tortuous and engorged retinal veins, and is frequently accompanied by spot- or flame-shaped retinal hemorrhages. Fundus angiography (FA) typically shows delayed retinal venous filling along with tortuous, dilated veins. The risk factors include hypertension, diabetes, dyslipidemia, and others, with the condition usually manifesting in older individuals. However, the pathogenesis of venous stasis retinopathy secondary to MOG-associated optic neuritis differs from typical cases. It is hypothesized that optic neuritis-induced optic nerve swelling may lead to impaired venous outflow, resulting in ischemic changes (23). In cases of MOG-associated optic neuritis with secondary venous stasis retinopathy, corticosteroid therapy has been shown to yield good visual recovery. To date, there have been two relevant case reports. Lukewich and Micieli (9) reported a 38-year-old woman with MOG-associated optic neuritis complicated by unilateral venous stasis retinopathy. Her vision deteriorated to counting fingers, accompanied by eye pain on movement. After 5 days of high-dose corticosteroid pulse therapy, her vision improved to 20/20. Srimanan and Ngathaweesuk (10) described a 27-year-old woman who presented with headache and fever for 2 weeks, followed by bilateral venous stasis retinopathy and severe preretinal hemorrhage in one eye. Her vision improved after systemic corticosteroid treatment.

The management of papillophlebitis predominantly involves glucocorticoid therapy, anti-VEGF therapy, or a combination of both modalities (24). Given that papillophlebitis is often associated with autoimmune processes, glucocorticoids are considered the first-line treatment. In cases where macular edema develops and demonstrates poor response to glucocorticoid therapy, anti-VEGF agents are recommended, as they are established as first-line therapy for macular edema secondary to CRVO (25). In the instance described herein, the patient underwent a regimen of combined anti-VEGF and glucocorticoid therapy for MOGAD, which led to a substantial enhancement in visual acuity. To the best of our knowledge, this represents the inaugural documentation of papillophlebitis as a sequela of optic neuritis in the context of MOGAD. When systemic glucocorticoid therapy alone proves inadequate in managing the condition, the timely integration of anti-VEGF therapy can result in superior visual outcomes. Although the pathogenesis in this case is not fully elucidated, it provides novel perspectives on the etiology and clinical ramifications of MOGAD. Furthermore, it underscores the necessity for clinicians to vigilantly observe for retinal alterations in patients undergoing treatment for MOGAD.

## Conclusion

Papillophlebitis is a rare ocular complication of MOG-associated optic neuritis. Regular fundus examinations are essential throughout the therapeutic regimen, particularly in younger patients. Prompt and appropriate therapeutic intervention can markedly enhance visual outcomes and avert complications in individuals afflicted with this condition.

## Data Availability

The datasets presented in this study can be found in online repositories. The names of the repository/repositories and accession number(s) can be found in the article/supplementary material.
